# *Strongyloides stercoralis*: Systematic Review of Barriers to Controlling Strongyloidiasis for Australian Indigenous Communities

**DOI:** 10.1371/journal.pntd.0003141

**Published:** 2014-09-25

**Authors:** Adrian Miller, Michelle L. Smith, Jenni A. Judd, Rick Speare

**Affiliations:** 1 Indigenous Research Unit, Griffith University, Brisbane, Australia; 2 Faculty of Medicine, Health and Molecular Sciences, James Cook University, Townsville, Queensland, Australia; 3 Public Health and Tropical Medicine, James Cook University, Townsville, Queensland, Australia; 4 Tropical Health Solutions Pty Ltd, Townsville, Queensland, Australia; Case Western Reserve University School of Medicine, United States of America

## Abstract

**Background:**

*Strongyloides stercoralis* infects human hosts mainly through skin contact with contaminated soil. The result is strongyloidiasis, a parasitic disease, with a unique cycle of auto-infection causing a variety of symptoms and signs, with possible fatality from hyper-infection. Australian Indigenous community members, often living in rural and remote settings, are exposed to and infected with *S. stercoralis*. The aim of this review is to determine barriers to control of strongyloidiasis. The purpose is to contribute to the development of initiatives for prevention, early detection and effective treatment of strongyloidiasis.

**Methodology/Principle Findings:**

Systematic search reviewing research published 2012 and earlier was conducted. Research articles discussing aspects of strongyloidiasis, context of infection and overall health in Indigenous Australians were reviewed. Based on the PRISMA statement, the systematic search of health databases, Academic Search Premier, Informit, Medline, PubMed, AMED, CINAHL, Health Source Nursing and Academic was conducted. Key search terms included strongyloidiasis, Indigenous, Australia, health, and community. 340 articles were retrieved with 16 original research articles published between 1969 and 2006 meeting criteria. Review found barriers to control defined across three key themes, (1) health status, (2) socioeconomic status, and (3) health care literacy and procedures.

**Conclusions/Significance:**

This study identifies five points of intervention: (1) develop reporting protocols between health care system and communities; (2) test all Indigenous Australian patients, immunocompromised patients and those exposed to areas with *S. stercoralis*; (3) health professionals require detailed information on strongyloidiasis and potential for exposure to Indigenous Australian people; (4) to establish testing and treatment initiatives within communities; and (5) to measure and report prevalence rates specific to communities and to act with initiatives based on these results. By defining barriers to control of strongyloidiasis in Australian Indigenous people, improved outcomes of prevention, treatment of strongyloidiasis and increased health overall are attainable.

## Author Summary

*Strongyloides stercoralis*, a nematode parasite, has a well-documented history of infecting human hosts in tropic and subtropic regions mainly through skin contact with inhabited soil. The result is strongyloidiasis, a human parasitic disease, with a unique cycle of auto-infection contributing to a variety of symptoms, of which, hyper-infection causing fatality may occur. In Australia, Indigenous community members often located in rural and remote settings, are exposed to and infected with strongyloides. Previous researchers report strongyloidiasis as a recurrent health issue for Indigenous Australians. This is a systematic review to determine the barriers to control for this pernicious pathogen. Barriers to control can be defined across three key themes: (1) health status, (2) socioeconomic status, and (3) health care literacy and procedure. By conceptualizing these barriers and addressing steps to control as outlined in this study, there is potential for improvement in prevention and treatment outcomes of strongyloidiasis and subsequently, overall health for Australian Indigenous people. This study contributes to furthering prevention and treatment of strongyloidiasis, increasing exposure to the issue of strongyloidiasis in Australian Indigenous people. It is the intent of this paper to express the need to have continued research and further health policy directed specifically to eradicate strongyloidiasis in Australian Indigenous communities.

## Introduction

*Strongyloidies stercoralis*, a nematode parasite, is well documented as a potentially fatal soil transmitted helminth, described as a unique and complex human parasite in Speare [1]. *S. stercoralis* is a cosmopolitan parasite, but is more prevalent in tropical regions of the world, including tropical Australia. Rural and remote regions of Australia, in particular, Queensland, Northern Territory, Western Australia, north of South Australia and northern areas of New South Wales, endemic rates [1]-[5]. Australia's Indigenous communities have high prevalence of strongyloidiasis (disease resulting from *S. stercoralis*) as do immigrants from other endemic countries, travellers to these countries and military personnel who have spent time in endemic regions [6], [7]. Soulsby, Hewagama and Brady [8] report four cases of strongyloidiasis in non-Indigenous people resulting from work-related exposure presenting at Alice Springs Hospital and by implication acquired indirectly from Indigenous populations. Those infected included a teacher at an Indigenous school, a child care worker, an ex-nurse and a paediatrician. Very high prevalence rates are reported for Australian Indigenous communities [3], [4], [6], [7], [9], [10]. Johnston, Morris, Speare, et al. [7] describe strongyloidiasis as a clinically important condition in Australia. Kline, McCarthy, Pearson, et al. [11] discuss major neglected tropical diseases in Oceania and emphasize strongyloidiasis as an important infection despite the lack of data on overall prevalence rates and clinical impact.

Strongyloidiasis in a community is evidence that individual(s) in that community has been exposed to *S. stercoralis* from soil contaminated by human faeces [6]. Infected individuals pass first stage larvae in the faeces; these develop on the soil to infective larvae which penetrate the skin of the next host. After a blood-lung migration, parasitic adult females (there is no parasitic male) molt and develop into adult female worms in tunnels in the small intestinal mucosa [12]. Eggs are then laid in the tunnels, hatch, and produce first stage larvae in the intestinal lumen. Most of these pass out in the feces. A small number, however, change to infective larvae in the gut. These autoinfective larvae penetrate the wall of the large intestine and re-enter the body. Hence, *S. stercoralis* is a very unusual nematode, producing infective larvae not only externally in the soil, but also internally [12].

The occurrence of the autoinfective larvae is the main reason strongyloidiasis is such a serious disease [12], [13]. Infection is life-long since adult worms are replaced by young worms and the infection does not end when the original crop of adults die. Worm numbers can rise incrementally to produce severe disease, known as the hyperinfection syndrome. Autoinfective larvae, migrating from the lumen of the large intestine, can carry enteric bacteria into the body, resulting in sepsis in any organ. Of patients with the hyperinfection syndrome, 50% present with a septic event (pneumonia, septicaemia, meningitis, peritonitis) usually caused by an enteric bacteria or polymicrobial suite of enteric bacterial [14]. Complicating this is that *S. stercoralis* has an immunosuppressive effect [15], [16]. Hyperinfection occurs mainly, but not exclusively, in the people who are immunocompromised or immunodeficient with a high case fatality rate of hyperinfection, at least 60% [6], [7], [9], [10], [13], [17], [18].

Strongyloidiasis is usually symptomatic [14] but most signs and symptoms are non-specific. The exception is with larva currens, a rapidly moving urticarial linear rash that marks the passage of an autoinfective larvae through the skin [14], [19]. This is pathognomonic of strongyloidiasis. The other non-specific signs and symptoms can include gastrointestinal (e.g., abdominal pain, nausea, diarrhea, weight loss), respiratory (e.g., cough (productive and non-productive), haemoptysis, cutaneous (e.g., urticara) and general malaise [7], [10], [14], [20]. Hyperinfective strongyloidiasis, in addition to the spectrum of acute-infection symptoms, can also clinically present as paralytic ileus, pulmonary haemorrhage, pneumonia, meningitis, septicaemia or other bacterial infections [6], [10], [14], [16], [18], [20]–[22].

Diagnostic testing includes serology and faecal examination. Once diagnosed, strongyloidiasis can be eradicated with specific anthelmintics, ivermectin being the drug of choice [6], [7], [12], [17]. The recommended treatment for strongyloidiasis has changed with the development of more effective anthelmintic drugs. Thiabendazole was the first moderately effective anthelmintic introduced in the mid-1970s [23], [24]. Albendazole, a benzimidazole like thiabendazole, was recommended as the treatment of choice for strongyloidiasis about the mid-1990s [25]. It was replaced by ivermectin as first line recommended anthelmintic in the early 2000s [10].

In Australia, ivermectin is not licensed for children <5 years or for use in pregnancy [26], [27], although there is no evidence of harm in these groups [10]. Albendazole is used for > 6 months and <10 kg to adults, not licensed for use during pregnancy [26]–[28]. Fatality from strongyloidiasis most often results from missed or late diagnosis, inadequate treatment and/or the use of immunosuppressant drug therapy in high risk groups [6], [10], [17]. Co-infection of strongyloidiasis with HTLV-1 is associated with more serious strongyloidiasis and potential resistance to treatment [10], [15]. In addition, HTLV-1 carriers are more likely to develop T-cell leukaemia when infected with *S. stercoralis* [29]–[32].

There are questions about the limited information available about the prevalence, clinical picture, diagnosis and public health approaches to manage strongyloidiasis in rural and remote Indigenous communities in tropical regions of Australia [5], [33]. Programs based on the treatment of stool positive individuals have also been associated with decreases in prevalence [7]. Researchers suggest that little published evidence of public health approaches to control strongyloidiasis exists [7], [34] and there is a need to consider mass drug administration in Indigenous Australian communities with high prevalence of strongyloidiasis [10], [11].

This systematic review attempts to answer the questions, what is the epidemiology of strongyloidiasis in Australian Indigenous people, and, what, if any, are the mentioned barriers to control? The aim of this review is to identify research focused on strongyloidiasis in this specific population and to collect and analyse available data specific to symptoms, diagnosis and treatment to determine barriers to control of strongyloidiasis. For the purpose of this paper, we respectively use the term Indigenous to represent Australian Aboriginal people and Torres Strait Islanders.

## Methods

The outline and focus of this paper is framed on the concept of a translational research framework described by Thomson [35] within the Australian Indigenous HealthInfoNet. This systematic review was designed as a narrative review of the evidence as a way to summarise, explain and interpret evidence with thematic analysis [36].

This systematic review was based on the PRISMA statement, a tool to summarize accurate, reliable, quality evidence by way of transparent reporting (Checklist S1) [37], [38]. A systematic search of health databases, Academic Search Premier, Informit, Medline, PubMed, AMED, CINAHL, Health Source Nursing and Academic was performed to search for all articles published 2012 and prior were included in the search. Articles were searched through the online academic search site, Google Scholar and internet searches for websites containing information about strongyloidiasis. Key search terms included strongyloidiasis, Indigenous, Australia, health, and community with search strategy developed to access the broadest range of articles about strongyloidiasis are presented in Table 1. Reference lists of original articles, review articles, grey literature and websites were searched for potential articles to review for inclusion. Language restrictions were not imposed.

**Table 1:** Search strategy.

Number	Keywords
1	Strongyloidiasis or strongyloides
2	Strongyloidiasis or strongyloides and Australia
3	Strongyloidiasis or strongyloides and Australia and Aboriginal or Indigenous
4	Strongyloid* and Australia
5	Strongyloid* and Indigenous
6	Strongyloid* and Indig*
7	Strongyloid* and Aborig* or Abor*
8	parasite infe* and Australia and Abor*
9	para* infe* and Australia and Abor*
10	para* infe* and Australia and Indig*
11	strongyloid* and community
12	strongyloid* and health
13	parasite and infe* and Australia and indig*
14	gastro* infe* and Australia and abor*
15	pedia* and Australia and abor*
16	infectious disease and Australia and abor*
17	11 and 4 or 5 or 6 or 7
18	12 and 4 or 5 or 6 or 7
19	10 and 16 and 5 or 6 or 7
20	1 and 16
21	5 or 6 and 15
22	10 and 11
23	10 and 12

*asterisks added to root word to find all forms of word during library search.

To meet inclusion criteria, original qualitative or quantitative research articles contained content addressing one or more of the following: symptoms, diagnosis, treatment, and barriers to control of strongyloidiasis. The location of the studies had to be Australia and include Australian Indigenous people. Exclusion criteria included, review articles and non-peer reviewed literature, original research articles with animal only studies, pharmaceutical therapy only studies and studies not differentiating *S. stercoralis* or strongyloidiasis from amongst other parasites or parasitic infections.

**Figure 1:**
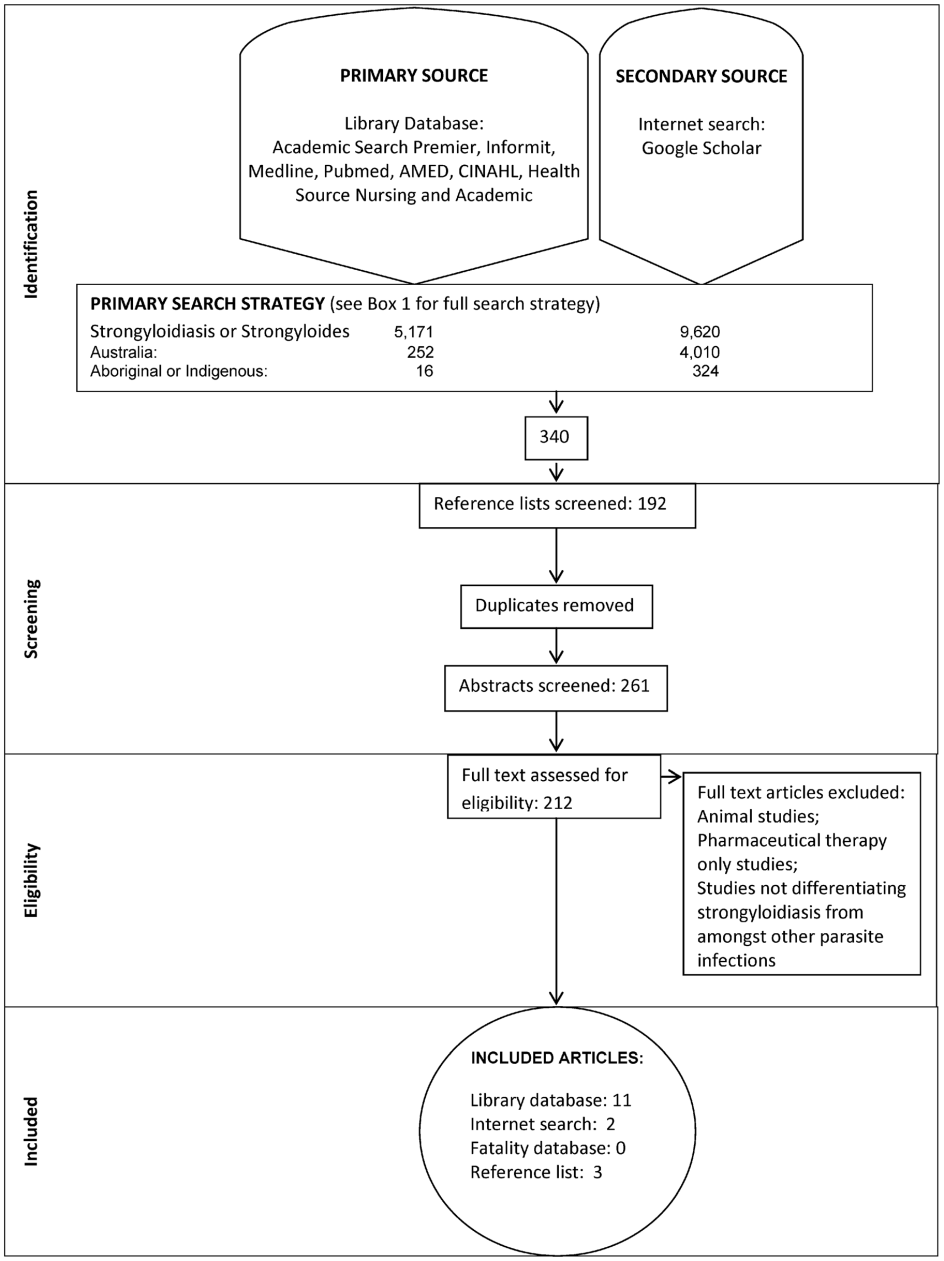
Flow diagram represents systematic review search based on the PRISMA statement reporting guidelines for systematic reviews and meta-analyses [38].

Based on these selection criteria, articles were reviewed in two stages. First stage, article titles and abstracts were screened to meet the requirements of strongyloidiasis as topic, Australian location and inclusion of Indigenous Australians. Second stage, articles were read as full text. Articles meeting final criteria were included in the study. Figure 1 represents the overall article search outcome.

From the original research questions, (1) what is the epidemiology of strongyloidiasis in Australian Indigenous people? and (2) what, if any, are the mentioned barriers to control? Description of studies was collected and a thematic analysis conducted [36]. Key data extracted were: purpose of study, study design, participant description, symptoms, diagnosis, treatment, barriers to control, and author's conclusions. Articles were presented in a database with publisher details and summarized key data. The categories of symptoms, diagnosis, treatment and barriers to control were further assessed and coded using thematic analysis to determine recurring items in each. Symptoms were defined as manifestations of strongyloidiasis and included symptoms and signs due to strongyloidiasis and other existing concurrent conditions. Diagnosis was defined medical diagnoses including health status, tests performed and results.

Assessment of treatment of strongyloidiasis was based on the recommended therapy at the time of publication and defined as details on therapy provided and the comments on outcomes. Barriers to control were defined as a medical context, symptom and/or condition, or social determinant (derived from categories of symptoms, diagnosis, treatment and each authors' summary and conclusions) that inhibited overall health and/or recovery from strongyloidiasis of the individual(s). Once the barriers to control items were documented, they were then coded into barrier themes and health level. Detailing each barrier and the associating theme and level supports the translational knowledge concept by assisting to identify the relevant stakeholders [39].

## Results

Figure 1 provides an overview of the literature search results. 340 articles were retrieved with a total of 16 articles, published between 1969 and 2006, eligible for the systematic review and are summarized in Table 2. Eleven eligible articles were from electronic library databases. Google Scholar revealed two additional eligible articles. The reference lists reviewed from published articles, grey literature and internet websites reporting on strongyloidiasis infections of Indigenous people of Australia revealed three eligible articles. Study design included case studies, retrospective and prospective comparison and non-comparison studies. Participant numbers ranged from 1 to 683. Indigenous Australian children were reported in 12/16 studies, of those 8/12 reported children only. Indigenous Australian adults were reported in 7/16 studies, of which 4/7 reported adult only. Thirteen studies were conducted in hospital and four in Indigenous communities. Eleven studies examined strongyloidiasis only with the remaining discussing the parasitic infection in the context of other infections [40], [41] or while examining gastrointestinal issues [42]–[44].The 16 papers included 2537 Indigenous participants and 272 non-Indigenous participants.

**Table 2:** Summary of publications with original research on strongyloidiasis in Australian Indigenous people*.

Study	Purpose of Study	Study Location	participants^+^	Study Design
[4]	To investigate the biomedical consequences of lifestyle changes among communities in order to help people understand changes and to cope with them.	Arhhem Land, Northern Territory	403 Iac	Cross-sectional and longitudinal
[5]	To report prevalence and distribution of infections with *S. stercoralis* in communities.	Remote communities, Queensland	122 Ic	Retrospective
[21]	To present the case of one adult with 10 episodes of meningitis due to strongyloidiasis.	Fitzroy Crossing, Western Australia	1 Ia	Retrospective case
[22]	To report a case study of a child that demonstrates how clinically unsuspected strongyloidiasis progresses to hyperinfection after increase in immunosuppression medication.	Adelaide Childrens Hospital	1 Ic	Case
[16]	To describe a case of hyperinfection.	Royal Darwin Hospital	1 Ia	Case
[28]	To explore the utility of antibody tests for confirming cure of strongyldoidiasis in endemic population.	Arnhem land, Northern Territory	508 Iac	Case control
[15]	To determine whether complicated strongyloidiasis occurs in association with HTLV-1 infection.	Alice Springs Hospital	18 Iac	Retrospective case
[41]	To compare infection-related mortality rates and pathogens associated for Indigenous and non-Indigenous adults.	Alice Springs Hospital	351 Ia; 162 Na	Retrospective comparison
[40]	To compare bloodstream infection rates, pathogens and mortality among Indigenous and non-Indigenous adults.	Alice Springs Hospital	614 Ia; 69 Na	Retrospective comparison
[42]	To report biopsy findings using histological assessment and examination under dissecting microscope in intestinal mucosal biopsies from children.	Royal Alexandra Hospital for children	30 Ic	Prospective comparison
[43]	To indicate the extent or severity of diarrheal disease in children in communities.	Kimberley region, Northern Territory	100 Ic	Prospective
[44]	To show that the severity of diarrheal disease in children as a consequence of underlying small intestinal mucosal damage.	Royal Darwin Hospital, Northern Territory	339 Ic; 36 Nc	Prospective comparison
[45]	To describe clinical presentation, diagnosis and management of strongyloidiasis and to identify predisposing factors.	Townsville General Hospital	9 Iac; 5 Nac	Retrospective
[46]	To describe strongyloidiasis in children.	Darwin Hospital	8 Ic	Case
[50]	To describe clinical and laboratory features of strongyloidiasis.	Royal Darwin Hospital	64 Iac; 4 Nac	Retrospective
[51]	To present the case of an infant with meningitis and who subsequently developed complete small-intestinal obstruction.	Royal Alexandra Hospital for Children	1 Ic	Case

+a = Adult(s); c = child(ren), ac = adult(s) and child(ren), I = Indigenous; N = non-Indigenous;

*For the purpose of this paper, we respectively use the term Indigenous to represent Australian Aboriginal people and Torres Strait Islanders.

Eleven papers described manifestations of strongyloidiasis, including symptoms and signs due to strongyloidiasis as well as other concurrent conditions (Table 3). Studies noted strongyloidiasis symptoms such as diarrhoea, malnutrition and anorexia, abdominal pain, abdominal distension, anemia, septicaemia, and fever. Other concurrent conditions including Type 2 Diabetes, Lupus, Chronic Liver Disease and Chronic Lung Disease, Alcoholism, Pneumonia, Bronchitis, COPD, Acute Rheumatic Fever, Acute Renal Failure and/or general gastrointestinal, cardiac and respiratory problems were reported. Gunzburg, Gracey, Burke, et al. [43] reported only diarrheal symptoms as this was the scope of the study. Page, Dempsey, and McCarthy [28] and Prociv & Luke [5], although studying strongyloidiasis specifically, did not focus on symptomology. Four studies [4], [15], [40], [42] did not discuss symptomology due to the aim of the study.

**Table 3:** Manifestations of strongyloidiasis in Indigenous Australian patients*.

Study	Participant details ^+^	Other condition	Symptoms/signs due to strongyloidiasis
[4]	403: 10 yr and older	hepatitis B	not listed
[5]	122: under 15 yr	not listed	not listed
[15]	513: 351 Ind; 162 Non	not listed	not listed
[16]	1 female 18 yr	Grade-IV lupus glomerulonephritis (LG) with nephrotic syndrome, hypertension, febrile neutropenia, chronic gastric erosions, non-insulin dependent diabetes, poor cardiovascular and respiratory function	diarrhea, abdominal pain, anorexia
[21]	1 male adult	recurring meningitis, alcoholism	*E. coli* septicaemia
[22]	1 female 12 yr	Systemic lupus erythematosus, paralytic ileus, candidiasis, pneumonia	anemia, headache, back pain, fever, confusion, bacterial septicaemia
[28]	508: 13 yr and older	not listed	not listed
[40]	614 Ind; 69 Non: under 15 yr	not listed	not listed
[41]	18 Case series (C) (4 detailed): C1 female 39 yr; C2 male 29 yr; C3 male 32 yr; C4 male 41 yr	C1 chronic liver disease, alcoholism, shoulder pain, epigastric pain, cachectic; C2 peripheral neuropathy, chronic liver disease, alcoholism, HTLV-1, hepatitis B, pleuritic chest pain, productive cough, dyspnea; C3 chronic liver disease, alcoholism, bilateral crackles, wheeze, dyspnea, hypotensive; C4 Type 2 diabetes, chronic liver disease, alcoholism, hypotensive, crackles, wheeze, acute renal failure, intravascular coagulopathy	C1 abdominal pain, severe pruritus, diarrhea, faecel incontinence; C2 abdominal pain, diarrhoea; vomiting, septic shock; C3 abdominal pain, pruritus, diarrhea; C4 Fever, diarrhea, abdominal pain
[42]	3: 1–5 yr	not listed	partial villous atrophy of third degree
[43]	100: 0–5 yr	not listed	Diarrhea
[44]	338 Ind; 37 Non: children	hypokalemia; cryptosporidium	diarrhoea; malnutrition
[45]	9 Case series: C1 17mos;C2 42 yr; C3 49 yr; C4 11yr; C5 7mo; C6 17 yr; C7 30 yr; C8 1 yr; C9 26 yr	C1 croup; C2 alcoholism, COPD, trichuriasis; C3 no details; C4 nil; C5 bronchitis, cryptosporidiosis; C6 alcoholism, trichuriasis; C7 systemic lupus erythematosus, alcoholism, giardiasis; C8 Giardiasis; C9 Alcoholism, trichuriasis, toxic epidermal necrolysis, allergies	C1 diarrhoea, rash; C2 abdominal pain; C3 no details; C4 diarrhoea; C5 diarrhoea; C6 abdominal pain, diarrhea, nausea, vomiting/C7 pruritus, death; C8 diarrhoea, vomiting, rash; C9 diarrhoea, septicaemia, recurrent infections
[46]	3 Case series: C1 1 yr; C2 2 yr; C3 4 yr	C1 anaemia; C2 bronchitis, otitis media; C3 acute rheumatic fever	C1 diarrhoea, failure to thrive, hypokalemia, hypernatremia, partial intestinal obstruction; C2 gastroenteritis, hypokalemia, partial intestinal obstruction; C3 gastroenteritis, intestinal obstruction
[50]	68: 64 Ind; 3 Non	Alcoholism, scabies (and “other” parasites), pulmonary disease, congestive cardiac failure	anaemia, diarrhea, gastrointestinal symptoms, malnutrition
[51]	1 female 6mo	Pneumonia, *H. influenza*, meningitis	Intestinal obstruction with granulomata around larvae, vomiting, abdominal distention
**Total**	**2537 Ind; 272 Non**		

+Participant details: Indigenous Australian unless otherwise specified, Ind = Indigenous, Non = non Indigenous.

*For the purpose of this paper, we respectively use the term Indigenous to represent Australian Aboriginal people and Torres Strait Islanders.

All sixteen studies provided data on diagnosis of strongyloidiasis determined by one or more tests (Table 4). Nine studies performed purposeful testing [4], [5], [21], [28], [40]–[43]. Five studies reported *strongyloidiasis* had been diagnosed when not suspected [15], [22], [42], [45], [46].

**Table 4:** Tests performed to diagnosis patients' condition not necessarily specifically related to strongyloidiasis diagnosis.

Study	Tests Performed
[4]	Blood; Stool
[5]	Stool
[15]	Abdominal scan; Chest x-ray; Serology; Stool
[16]	Abdominal scan; Brain scan; Chest x-ray; Blood; Stool
[21]	Cerebral spinal fluid protein level/neutrophil count; CT scan; Blood; Stool
[22]	Cytology; Gastric aspirate; Lung biopsy
[28]	Serology
[40]	Blood
[41]	Serology
[42]	Intestinal biopsy
[43]	Stool
[44]	Blood; Stool
[45]	Stool
[46]	Abdominal x-ray; Chest x-ray; Gastric aspirate
[50]	Stool
[51]	Abdominal x-ray; Abdominal x-ray/barium enema; Gastric aspirate; Laparotomy; Lumbar puncture; Stool

Articles were reviewed for the adequacy of treatment noting that recommended therapy has changed with time (Table 5). Eight articles discussed the use of one or a combination of albendazole, thiabendazole and ivermectin. Three articles described a subgroup of patients receiving no therapy [28], [42], [45] and one article mentioned the use of pyrantel only for strongyloidiasis [5]. Pyrantel is ineffective against *S. stercoralis* [47]. In two articles, prednisolone or prednisone, a treatment which suppresses the immune system and as a result can increase the severity of strongyloidiasis, was administered to patients. Walker-Smith [42] discussed diagnoses of giardiasis and strongyloidiasis in children and provided no data on treatment. Einsiedel & Fernandes [15] detailed treatment therapies across four case studies, of which, only one case received correct strongyloidiasis treatment with ivermectin. Overall, adequate treatment was documented in publications in only 5.2% of cases.

**Table 5:** Assessment of whether cases reported in papers were adequately treated according to the recommended anthelmintic for that time.

Study	Anthelmintic used	Comment	Total	Evidence*	%
[4]	No comment on treatment	Total 411 (positive: 60% serology; 41% faeces)	246	0	0
[5]	Pyrantel used as a routine de-wormer in Queensland Aboriginal health program – does not treat strongyloidiasis; thiabendazole given for strongyloidiasis (sometimes) but usually for 2 days not 3; so arguably none received adequate treatment	Multiple cases in children (<16yr) – 1971–1991: thiabendazole used, but probably not for most cases; comment made that children often refused drug due to unpleasant side effects	632	0	0
[15]	Albendazole = 1 (single dose); Ivermectin = 3; No treatment = 14	In 18 patients treatment was inadequate since 14 no treatment; 1 single dose albendazole; 3 single dose of ivermectin. (15/18 patients died)	18	0	0
[16]	Albendazole and ivermectin (sequence)	Treatment successful	1	1	100
[21]	No comment on therapy	1 adult male	1	0	0
[22]	No comment	Indigenous female child with hyperinfection	1	0	0
[28]	Albendazole single = 10 (inadequate); Albendazole multiple = 10 (adequate); Ivermectin single = 19 (inadequate); Ivermectin multiple = 42 (adequate)	Was a critical paper in that demonstrated albendazole was less effective than ivermectin; hence, both albendazole and ivermectin considered adequate	79	52	66
[40]	No comment	Study on blood stream infection	73	0	0
[41]	No comment	Study on deaths in hospitalized patients	2	0	0
[42]	None described	Not stated how many children had *S. stercoralis*			
[43]	No comment on treatment	12 children with *S. stercoralis* in faeces	12	0	0
[44]	No comment	Study on diarrhoea in children admitted to Royal Darwin Hospital	23	0	0
[45]	Thiabendazole	Of 6 adults, 4 adequately treated; Of 3 children, 2 adequately treated	9	6	67
[46]	Thiabendazole	Case 1: 1 course of unstated length; eosinophilia on discharge; Case 2: No details; eosinophilia on discharge; Case 3: No details	3	0	0
[50]	Thiabendazole	Details for Indigenous patients not given; comment made that 57% of all (not just Indigenous) patients received adequate treatment	64		57 (54–61)
[51]	Thiabendazole multiple doses and courses	No larvae found at end and eosinophil count normal	1	1	100
**Total**			**1165**	**60**	**5.2**

*Evidence of adequate treatment.

Barriers to control of strongyloidiasis were summarized in terms of item, theme and health access level (Table 6). Three barriers themes emerged as items contributing to adequate management of strongyloidiasis: (1) health status; (2) socioeconomic status; (3) health care literacy and procedures. Theme 1, health status was defined patients' health prior to and at the time of diagnosis of strongyloidiasis. This included concurrent infections (e.g., meningitis, pneumonia), concurrent chronic health conditions (e.g., Lupus, Chronic Liver Disease, Chronic Lung Disease, Acute Rheumatic Fever, HTLV-1, Hepatitis B, alcoholism, immunocompromised, immunosuppressed) and the phenomenon of strongyloidiasis (e.g., re-infection, hyperinfection, at times asymptomatic, chronic diarrhoea, septicaemia). Theme 2, socioeconomic status included living conditions, racial disparities, communication (e.g., interaction between community, patients, health professionals/institutions).Theme 3, health care literacy and procedures involved barriers that influence the diagnosis and treatment outcomes (e.g., delayed diagnosis, difficult to detect, failure to recognize symptoms, inadequate knowledge/treatment/treatment dose, serology test cut off, lack of communication, lack of screening, lack of follow-up, treatment non-compliance).

**Table 6:** Barriers to control of strongyloidiasis.

Item described in one or more studies	Barrier Theme^+^	Level*	[4]	[5]	[15]	[16]	[21]	[22]	[28]	[40]	[41]	[42]	[43]	[44]	[45]	[46]	[50]	[51]
Antibiotic prior treatment	(1)(3)	(1)(2)(3)(4)								Y								
Chronic Diarrhoea	(1)(3)	(1)														Y		
Concurrent Chronic infections	(1)(2)(3)	(1)(2)(4)					Y	Y										
Concurrent Health Conditions/Disease	(1)(3)	(1)(2)(3)			Y	Y	Y									Y		
HTLV-1	(1)(2)	(1)(2)(3)(4)			Y													
Immunocompromised	(1)(3)	(1)(3)						Y										
Immunosuppression	(1)(3)	(1)(3)			Y	Y												
Sepsis	(1)(3)	(1)(3)			Y		Y											
Malignancy	(1)	(1)			Y													
Malnutrition	(1)(2)	(1)(2)(4)			Y									Y		Y		
Hypokalemia	(1)	(1)(3)												Y				
Hyperinfection	(1)(3)	(1)(3)(4)			Y	Y	Y	Y										
Re-infection	(1)(2)(3)	(1)(2)(3)(4)					Y		Y	Y								
Asymptomatic	(1)(3)	(1)											Y			Y		
Delayed Diagnosis	(2)(3)	(3)(4)			Y	Y		Y							Y			
Difficult to detect	(1)(2)(3)	(3)(4)						Y										
Failure to Recognize Symptoms	(3)	(3)(4)			Y		Y											
Inadequate Knowledge	(3)	(3)(4)		Y	Y		Y					Y						
Inadequate Treatment	(3)	(3)(4)			Y	Y						Y					Y	Y
Inadequate treatment dose	(3)	(3)(4)		Y														Y
Serology test cut off	(3)	(3)(4)							Y									
Lack of Communication	(2)(3)	(2)(3)(4)			Y													
Lack of screening	(3)	(2)(3)(4)																Y
Lack of/Inadequate Follow-up	(2)(3)	(1)(2)(3)(4)			Y										Y		Y	
Treatment Non-compliance	(1)(2)(3)	(1)(2)(3)(4)		Y			Y								Y			
Racial Disparities	(2)(3)	(1)(2)(3)(4)									Y							
Lower SES	(1)(2)(3)	(1)(2)(4)	Y				Y			Y								
Living conditions	(1)(2)	(1)(2)(4)	Y				Y			Y	Y			Y				

Y = at least one incident of symptom or condition or determinant reported in one or more patients.

+(1) Prior/current health status; (2) Overall SES status; (3) Health care knowledge and procedures.

*(1) Individual; (2) Public/Community; (3) Organization; (4) Healthcare system.

Einsiedel & Fernandes [15] had the largest number of symptoms and signs and other conditions associated with barriers to control of strongyloidiasis. The top four barriers listed most often (determined by the most barriers per article, total of 4) were delayed diagnosis, inadequate treatment, living conditions and malnutrition. Barriers to control are located across all four health access levels: (1) Individual; (2) Public/Community; (3) Organization; and (4) Healthcare system.

## Discussion

This study reviewed original articles on strongyloidiasis in Indigenous Australian people. Articles were analyzed for symptoms, diagnosis and treatment and barriers to control of Strongyloidiasis. Overall outcomes are presented as *symptomology, diagnosis and treatment protocols, community research and action and addressing barriers to control*.

### Symptomology

The broad spectrum of symptoms, as represented in manifestations of strongyloidiasis in Table 3, illustrates the complex nature of Strongyloidiasis that is so often misdiagnosed. Many of these manifestations, such as diarrhoea, stomach pain, malnutrition, dehydration and vomiting are common to many illnesses and diseases. As described by researchers [6], [15], [16], [20], [43], [45], [46], strongyloidiasis can present many varying symptoms or be asymptomatic [43], [46]. It is important to recognize that strongyloidiasis can potentially exist for years presenting often with non-specific symptoms and signs (e.g., diarrhoea) as well as at times with periods without symptoms.

#### Hyperinfection

Einsiedel and Fernandes [15], Byard, Bourne, Matthews et al., [22] and Potter, Stephens and De Keulenaer [16] report specific cases of hyperinfection. Of these 4 specific cases fatality occurred in two of these studies [15], [22]. Results support previous research indicating that cases of hyperinfection and fatality may be prevented the earlier strongyloidiasis is diagnosed as undetected strongyloidiasis over longer periods lead to this outcome. Adams, Page and Speare [6] and Speare and Durrheim [12] report attention must be paid to those who are immunocompromised and, in all cases, steroid medication should not be administered until a diagnosis of strongyloidiasis is confirmed or ruled out. Early diagnosis increases probability of recovery. The possibility of hyperinfection or disseminated strongyloidiasis in immunocompromised patients, particularly in endemic areas, needs consideration [48]. The current protocol in place is to give the first dose of ivermectin when strongyloidiasis is suspected (i.e., when blood or faeces is taken) and then to give follow-up doses when test are positive. For those from a high prevalence area taking an immunosuppressive treatment (and until finished) are to continue with follow up strongyloidiais treatment every three months [26], [27], [49].

### Diagnosis and treatment protocols

Delayed diagnosis, inadequate knowledge/treatment/treatment dose, lack of communication and lack of follow up by health professionals were described as particular issues in the majority of studies [5], [15], [16], [22], [29], [40], [44], [45], [50], [51]. Infection should be suspected in every person with unexplained abdominal pain, diarrhoea, cutaneous symptoms or eosinophilia and the laboratory alerted of a provisional diagnosis [45]. Testing for strongyloidiasis is particularly important for patients from populations in *S. stercoralis* endemic areas. Rural and remote Indigenous communities (more specifically northern Australia) and including immunocompromised patients are at particular risk for hyperinfecion before administering immunosuppressive medication [22]. Protocol including clinical screening index, stool microscopy and culture, full blood count, immunoglobulin levels, and serological testing is recommended [22].

Majority of studies reported Indigenous Australian children with strongyloidiasis suggesting a diagnosis of strongyloidiasis should be considered when Indigenous children presenting with even non-suspecting general gastro-intestinal symptoms. Mucosal damage in Indigenous Australian children is possibly a result of damage produced by repeated episodes of gastroenteritis and/or parasitic infection, including strongyloidiasis [42]. Reduction in the frequency of gastroenteritis and parasitic infection in Indigenous children should greatly reduce incidence of small intestinal mucosal damage [42]. Working to eradicate or reduce strongyloidiasis infection in children with early detection and immediate treatment could decrease strongyloidiasis and mucosal damage. Given the challenges of diagnosing infection, standardizing treatment in communities for an extended period could potentially decrease infections rates [5].

#### Lack of follow-up

There was a repeated lack of follow-up within and across cases of strongyloidiasis [15], [45], [50]. It is quite possible that patients treated for stronygloidiasis may continue to carry the infection as has been presented in cases with people suffering from strongyloidiasis infection for years after initial exposure [16], [21]. This is problematic for a number of reasons. There is increased health risk to the patient as a result of continued infection including hyperinfection and fatality. The lack of awareness of continued infection in patient leads to increased risk for infection in the patients' community and decreases awareness by health professionals and community for need to eradicate the infestation within community and finally. This leads to inadequate reporting of strongyloidiasis in communities and under-representation of strongyloidiasis prevalence rates. Diagnosis and treatment of strongyloidiasis is challenging and requires specific knowledge. This knowledge must be acquired and maintained by health professionals in Australia and in particular, when assisting Indigenous Australian community members [6]. Assistance begins not only at the point of care in the hospital but also at the community level.

#### Treatment

The low rate of adequate treatment documented in the cases reported in the literature is of concern (Table 5). Einsiedel and Fernandes [15] highlighted that many (14/18) Indigenous patients in Central Australia received no treatment. Our reassessment of the four patients that did receive treatment in their series showed that all regimes were inadequate. Serological diagnosis means that confirmation of strongyloidiasis is usually delayed and for patients in remote areas of Australia this delay may have extended to several weeks [12]. As a result some clinicians used the approach that if a sample was collected for *S. stercoralis* serology the patient should receive the first dose of ivermectin [48]. Subsequent management would then depend on the serological result.

### Community research and action

Parasitic diseases have significant health risk and morbidity for Australian Indigenous people [11], [20]. Rural and remote communities are the most affected [3], [18]; mainly in children; and those immunocompromised with a number of cases of fatality reported [15], [22], [40], [41]. Studies in 2002 and 2005 report there are limited published examples of community interventions in Australia to control strongyloidiasis [7], [52]. Johnston, Morris, Speare, et al. [7] found no evidence of studies examining roles of environmental interventions and expressed the need to do so. The need for initiatives for housing and sanitation are imperative [15]. Issues of environmental health must be addressed concurrently with health service initiatives to develop long term and sustainable improvements in control of infectious parasitic and non-parasitic diseases in rural and remote Indigenous communities in Australia [10], [11], [20]. There may be increased risks associated with a casual approach to management and may be significantly higher for Indigenous Australian people living in HTLV-1 endemic Central Australia [10], [40]. Einsiedel and Woodman [40] further state the risk of strongyloidiasis in Indigenous communities and HTLV-1 infection may further predispose people to complicated strongyloidiasis.

### Addressing barriers to control

Steps to address the barriers to control should include: (1) development of *S. stercoralis* and strongyloidiasis reporting protocols across health care system and communities (e.g., consistent case study reporting methods, documentation of current infection sites) [6], [40]; (2) testing all Indigenous Australian patients, immunocompromised patients and those exposed to or living in areas of strongyloidiasis (e.g., rural/remote communities) presenting with gastrointestinal or respiratory symptoms (take particular notice of individuals from these groups with repeated visits to hospital) [7], [15], [16], [48]; (3) requirement of health professionals to have detailed information and education regarding strongyloidiasis and the potential for exposure in Indigenous Australian communities (e.g., understanding of the expanse of symptoms and potential for asymptomology, difficulty in diagnosis, need for variety of tests and retesting, accurate follow-up to confirm patient cleared of infection) [5], [15], [21], [42]; (4) establishment of testing and treatment initiatives in the community (e.g., over extended periods and periodically and treat symptomatic and asymptomatic strongyloidiasis carriers) [6], [10], [12], [15], [45]; (5) measure and report prevalence specific to Indigenous Australian communities and to act with initiatives based on these results [6], [12], [40].

#### Limitations

Studies analyzed for this review had an overall lack of detailed information on prevalence rates, diagnosis and treatment outcomes. Repeated lack of follow-up made it difficult to determine outcomes for those reported infected with strongyloidiasis in studies. In addition, a number of articles [5], [15], [50] conducted retrospective studies of hospital records with reported missing data, missing records and inconsistent reports. Case studies did not have a consistent reporting protocol to facilitate analysis within and across cases. It was unfortunate that a number of studies had to be excluded from this review as they had gathered overall parasite infection data in Indigenous Australian communities but had not further represented data by parasite (e.g., hookworm, *S. stercoralis*). This data would have been potentially valuable for increasing both the evidence and support to further define strongyloidiasis a problem for Indigenous Australians.

#### Conclusions

If barriers are managed, current research and the health care system can report accurately and provide the data required to support initiatives to eradicate strongyloidiasis in Indigenous Australian communities. Addressing these barriers would support conclusions of researchers that health education and public health interventions and guidelines for mass treatment with follow-up for effective treatment are essential [6], [10], [11]. As Einsiedel and Woodman [40] state sustainable improvements require a coordinated approach based on dialogue, cultural understanding and development of locally specific solutions by Indigenous people themselves. This comprehensive focus with Indigenous Australian people and their communities on strongyloidiasis is imperative. Community initiatives to eradicate endemic parasite infection such as hookworm have had success and there is potential to do the same with *S. stercoralis* [10].

## Supporting Information

Checklist S1 PRISMA 2009 checklist [38] utilized in systematic review with referring page numbers, tables and figures represented in manuscript.
(DOC)
